# Movement of the human foot in 100 pain free individuals aged 18–45: implications for understanding normal foot function

**DOI:** 10.1186/s13047-014-0051-8

**Published:** 2014-11-28

**Authors:** Christopher J Nester, Hannah L Jarvis, Richard K Jones, Peter D Bowden, Anmin Liu

**Affiliations:** School of Health Sciences, University of Salford, PO 32 Brian Blatchford Building, Salford, M6 6PU UK

**Keywords:** Normal foot kinematics, Foot motion, Foot segments, Foot model

## Abstract

**Background:**

Understanding motion in the normal healthy foot is a prerequisite for understanding the effects of pathology and thereafter setting targets for interventions. Quality foot kinematic data from healthy feet will also assist the development of high quality and research based clinical models of foot biomechanics. To address gaps in the current literature we aimed to describe 3D foot kinematics using a 5 segment foot model in a population of 100 pain free individuals.

**Methods:**

Kinematics of the leg, calcaneus, midfoot, medial and lateral forefoot and hallux were measured in 100 self reported healthy and pain free individuals during walking. Descriptive statistics were used to characterise foot movements. Contributions from different foot segments to the total motion in each plane were also derived to explore functional roles of different parts of the foot.

**Results:**

Foot segments demonstrated greatest motion in the sagittal plane, but large ranges of movement in all planes. All foot segments demonstrated movement throughout gait, though least motion was observed between the midfoot and calcaneus. There was inconsistent evidence of movement coupling between joints. There were clear differences in motion data compared to foot segment models reported in the literature.

**Conclusions:**

The data reveal the foot is a multiarticular structure, movements are complex, show incomplete evidence of coupling, and vary person to person. The data provide a useful reference data set against which future experimental data can be compared and may provide the basis for conceptual models of foot function based on data rather than anecdotal observations.

## Background

Characterisation of motion in the normal healthy foot is a prerequisite for understanding the effects of pathology on foot function and setting targets for mechanical interventions such as orthoses. In addition, quantitative data describing normal foot motion (and other biomechanical characteristics such as plantar pressure) should be the basis for conceptual clinical models of foot function. This contrasts with existing clinical models which are based largely on theory [1-7].

Defining the location (where motion occurs), the extent (magnitude) and the nature (e.g. timing of motion events or coupling) of normal foot motion requires that we have appropriately comprehensive and trustworthy data from feet of healthy individuals. However, most literature describing normal foot motion is limited by the small number of segments of the foot investigated, small sample size, or both. The most valid kinematic data requires direct measurement of bone motion [8] or imaging of individual bones [9,10]. However these inevitably suffer from a lack of generalisability. Use of skin mounted markers enables larger population studies, but the compromise is reduced validity due to skin movement artefact.

Many reports provide incomplete descriptions of foot motion. The largest study of foot motion to date (n = 153) [11] reported only leg, calcaneus, navicular and first metatarsal motion, ignoring lateral foot structures entirely. Indeed, how to best represent the functional units of the foot is still unresolved, with considerable variation in practice see [12,13]. Combining too many joints into a single rigid segment can lead to incorrect conclusions about where motion is occurring. For example, Jenkyn *et al.* [14] reported on sub talar kinematics using data of heel motion relative to the leg, whereas this movement is a result of the combined ankle and sub talar joints. Hunt *et al.* [15] reported that the forefoot contributed less motion than the rearfoot on the basis of combining 10 forefoot bones into a single forefoot segment. Subsequent research has shown that rear, mid and forefoot joints demonstrate similar amounts of motion during walking and thus require separate consideration [8]. Indeed, motion on the lateral arch of the foot (calcaneus-cuboid-fifth metatarsal) has been shown to equal that of the more commonly reported medial arch [8,16] and thus warrants separate reporting. However, to date kinematic data for separate medial and lateral forefoot segments is limited to small samples (n < 12) [17-19].

Having all the appropriate functional units in the foot represented in a multi segment foot model is also critical to investigation of how different joints contribute to the overall sagittal, frontal and transverse plane behaviour of the foot. The different contributions to walking by rear, mid, medial or lateral forefoot segments and toes, and coupling relationships between segments, might all change during gait. This seems highly likely since there is clear evidence of variation during gait in the ground reaction (e.g. centre of pressure path) and muscles forces (e.g. EMG data) input to the foot [20].

Prior literature on normal foot motion has focused on stance phase because external load clearly has a strong influence on tissue stress and injury. However, events during the contact phase will be influenced by events in late swing. Caravaggi *et al.* [21] proposed that late swing foot motion pretensions the plantar structures and influences the load acceptance and resistance to external pronation moments after initial contact. Understanding the events post toe off and prior to heel strike may therefore still offer insight into stance phase foot biomechanics.

To address the need for appropriately comprehensive quantitative foot kinematic data in a sufficiently large pain free population, we sought to: describe 3D foot kinematics for the leg, calcaneus, midfoot, medial forefoot, lateral forefoot, and hallux during stance and swing in a large population. Our purpose was to define normal kinematic patterns across a full range of functional units in the foot and to better understand how different joints contribute to the overall role of the foot during walking. This can provide an improved basis for defining normal foot kinematics in clinical models and future research studies.

## Methods

### Participants

Ethical approval for the research was granted by the University of Salford ethics committee and all participants provided written consent. Through advertising, introductory presentations and workshops 140 asymptomatic and otherwise self reported healthy individuals aged 18–45 were recruited from a University student and staff population. Medical history (including current and prior medication), vascular assessment (palpation of foot pulses), neurological assessment (vibration perception using 128Hz fork, light touch perception using 10 g monofilaments), and calculation of BMI were undertaken. Participants were excluded if they had prior history of musculoskeletal disease, foot or lower limb pain in last 6 months, had BMI < 16 or >30, had worn foot orthoses previously, and presented with any sign of compromised vascular or neurological status. Participants were excluded if either foot displayed hallux-abducto valgus indicated as lateral deviation of the hallux and medial metatarsal prominence. Screening identified 100 participants (mean age 31 (SD = 15.4, Range =18-45), 71 female, mean body mass 71.8 kg (SD =14.0, Range 47–107), height 168.3 m (SD = 8.1, Range =153-188).

### Salford foot model

**Figure 1:**
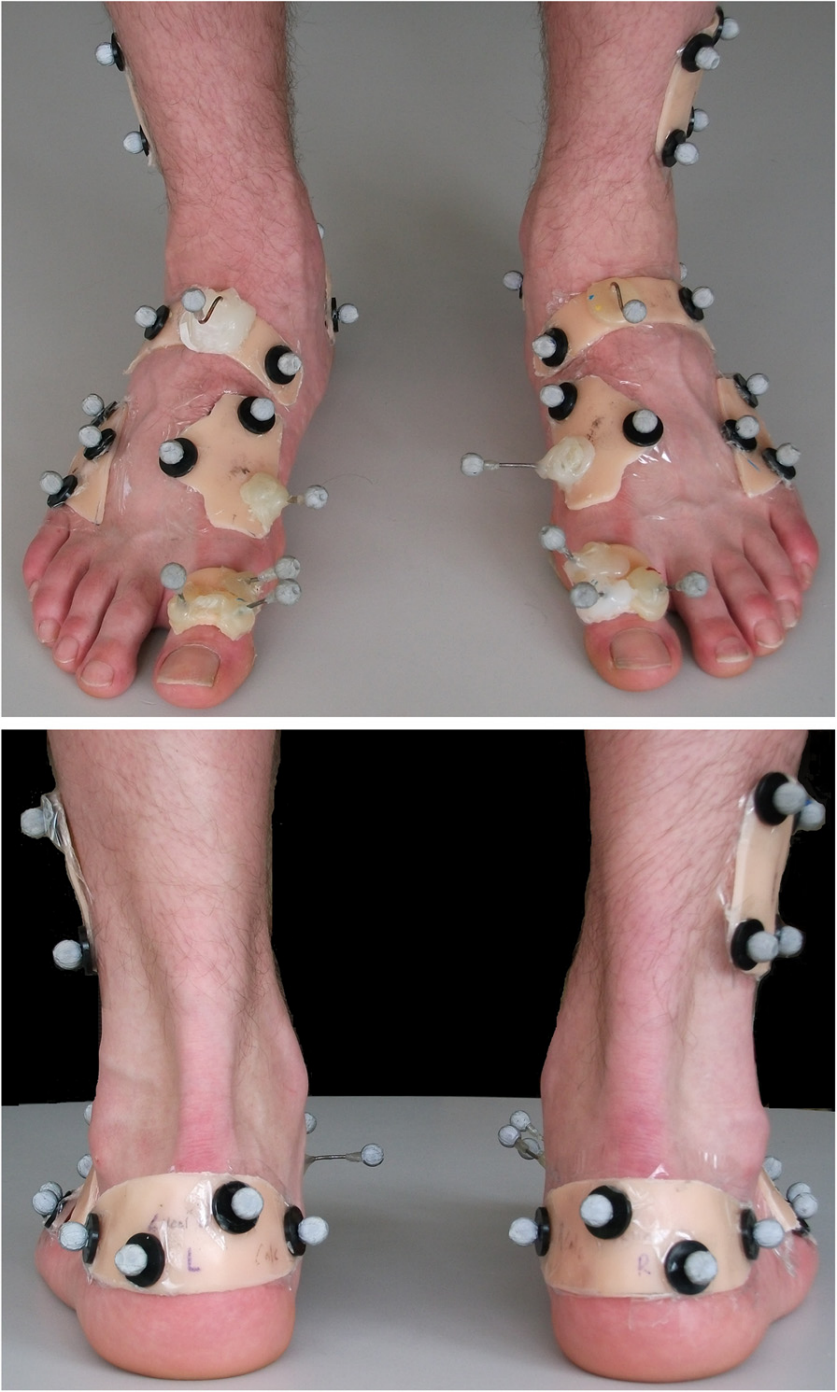
**Marker and plate locations for the multi segment foot model.**

A 6 segment model (leg, calcaneus, midfoot (navicular and cuboid), lateral forefoot (fourth and fifth metatarsals), medial forefoot (first metatarsal) and hallux was used to characterise foot kinematics. Rigid plastic plates were heat moulded to plaster casts of size 4 and 6 female feet, and sizes 9 and 12 male feet to enable improved fitting for different foot sizes. Each plate had three or four 7 mm markers attached (Figure 1). Placement of plates on appropriate underlying bones was assisted through manual palpation and manipulation of adjacent joints (e.g. flexing/extending the fifth metatarsal to establish the location of the cuboid-metatarsal joint).

### Data collection

Kinematic data were collected using 12x100 Hz cameras (Qualisys, Sweden). Force plate data (AMTI, 1500Hz) was collected to determine the start and end of stance/swing. Subjects walked at their own self select speed and 8 walks were recorded. A standing reference trial was collected to define 0° in the kinematic data. During the standing trial anatomical markers were placed on medial and lateral knee joint margins and the medial and lateral malleoli.

### Data processing

Kinematic data were processed in Visual3D and low pass filtered (6 Hz, Butterworth). For each of the five foot segments and the leg a local co-ordinate system (LCS) was defined using the reflective markers. The vertical (z) axis of the leg LCS was a line joining the midpoint of the malleoli distally, and midpoint of the medial and lateral knee margins proximally. The anterior/posterior axis (y) was determined by the unit vector perpendicular to the frontal plane that was a least squares plane through the z axis and the four anatomical markers on the knee and malleolus. The medial/lateral (x) axis was perpendicular to z and y. The foot segment LCS axes were all set parallel to those of the leg LCS during the standing reference trial.

Angular motion was calculated for 5 inter-segment combinations that were assumed to have 6 degrees of freedom: Calcaneus-tibia, midfoot- calcaneus, medial forefoot-midfoot, lateral forefoot-midfoot and hallux-medial forefoot (Cardan sequence x-y-z). The mean of 8 walking trials was derived.

### Data analysis

The peak + ve and –ve angular values during stance, angles at initial contact (IC), forefoot loading (FFL), toe off (TO), and heel off (HO), and the range of motion during swing were derived. These were thought to provide overall characterisation of foot kinematics at key events and be of use to future studies and clinical models of foot function that might use the data presented. The mean and 95% confidence intervals for the parameters were calculated in SPSS from one side (left).

Timing of IC and TO were defined using vertical ground reaction force data. The second heel strike (after swing phase) was defined using target pattern recognition [22]. FFL and HO were derived using changes in sagittal plane kinematics of a whole foot segment relative to the leg, following Richards [23]. This whole foot segment was defined in Visual3D using the malleoli midpoint proximally and the most distal marker on each of the medial and lateral forefoot segments. The whole foot angular rotations were expressed in the LCS of the leg. FFL was assumed to occur when contact phase plantarflexion of the foot-leg ceased [24]. Following Lundgren *et al.* [8], HL was assumed to coincide with maximum foot-leg dorsiflexion.

To explore how different foot segments contribute to the overall behaviour of the foot in each plane, the motion of each segment (e.g. calcaneus-tibia) was expressed as a% of the total motion occurring in that plane. The total motion in each plane was derived by summing all the motions at each of the 5 foot segment combinations for each% of the gait cycle, independent of the + ve or –ve sign.

## Results

**Figure 2:**
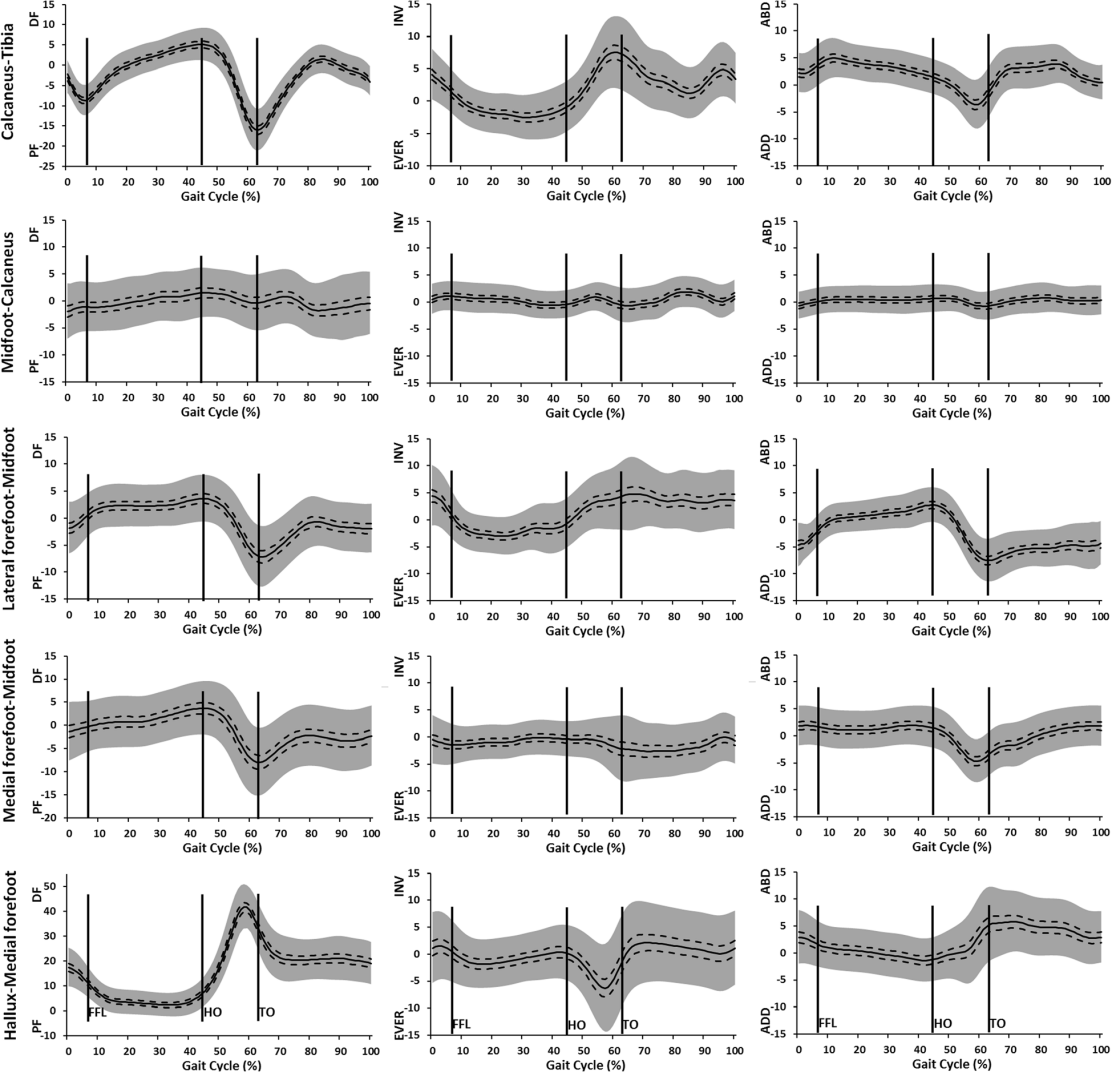
**Mean kinematic data (°) (+/− 95 CI (dashed line) +/− 1SD (grey)) for calcaneus-tibia, midfoot-calcaneus, lateral forefoot-midfoot, medial forefoot-midfoot and hallux-medial forefoot segments to the total motion occurring at each instant of the gait cycle.** +ve contributions are dorsiflexion (DF), inversion (INV) and abduction (ABD) of the distal segment relative to the proximal segment. 0° = position in relaxed standing.

**Figure 3:**
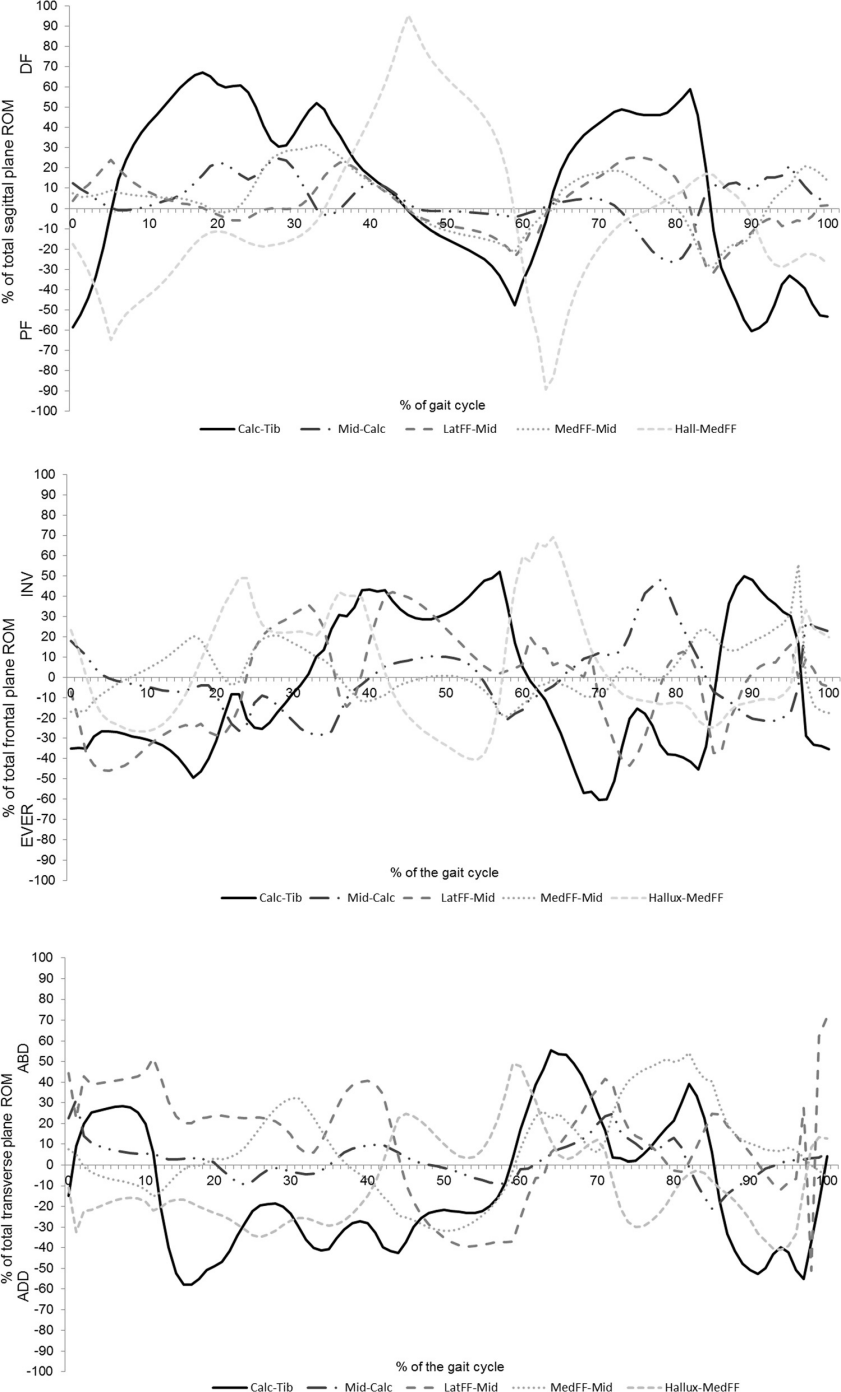
**Contribution by the calcaneus-tibia (calc-tib), midfoot-calcaneus (mid-calc), lateral forefoot-midfoot (latFF-mid), medial forefoot-midfoot (medFF-mid) and hallux-medial forefoot (hallux-medFF) segments to the total range of motion occurring at each instant of the gait cycle.** +ve contributions are dorsiflexion (DF), inversion (INV) and abduction (ABD) of the distal segment relative to the proximal segment.

The kinematics of the foot segments during gait are illustrated in Figure 2. Contribution by each segment combination to the total motion in each plane is illustrated in Figure 3. The kinematic values for specific events during gait are detailed in Table 1.

**Table 1:** **For each foot segment combination and plane of motion, mean (95% CI) of the angle of at IC, FFL, HO and TO, the maximum and minimum angles in stance, and the total range of motion during swing**

		**Angle at:**								**Max (Stance)**		**Min (Stance)**		**ROM swing**	
		**IC**		**FFL**		**HO**		**TO**							
		***Mean***	***95% CI***	***Mean***	***95% CI***	***Mean***	***95% CI***	***Mean***	***95% CI***	***Mean***	***95% CI***	***Mean***	***95% CI***	***Mean***	***95% CI***
Calcaneus-tibia	*Sag*	−3.1	−3.9– –2.2	−8.9	−9.8– –8.2	5.6	4.8– 6.4	−17.2	−18.3– –16.1	5.8	4.9– 6.6	−17.1	−18.2– –16.1	19.5	18.4– 20.5
	*Frt*	4.0	3.2– 4.8	1.9	1.3– 2.7	−0.9	−1.6– −0.6	6.8	5.5– 8.1	8.9	7.9– 9.9	−3.9	−4.7– –3.3	−5.1	−6.7– –3.5
	*Trn*	2.2	1.5– 2.9	3.1	2.3– 3.9	1.9	1.3– 2.6	−1.8	−2.8– –0.8	6.4	5.7– 7.0	−5.0	−5.9– –4.2	3.6	1.9– 5.3
Midfoot–calcaneus	*Sag*	−2.1	−3.2– –1.1	−1.2	−2.1– 0.2	1.6	0.6– 2.5	−0.5	−1.6– 0.6	3.2	2.3– 4.1	−2.8	−3.8– 1.9	−1.8	−2.4– –1.3
	*Frt*	0.5	−0.1– 1.1	1.1	0.5– 1.6	−0.5	−1.0– 0.7	−0.5	−1.2– 0.1	1.9	1.4– 2.5	−1.7	−2.3– 1.1	3.1	2.1– 4.0
	*Trn*	−0.7	−1.2– –0.2	0.0	−0.5– 0.5	0.6	0.1– 1.1	−0.8	−1.4– 0.3	1.5	1.0– 2.1	−1.5	−2.0– –0.9	0.5	−0.3– 1.3
Lateral forefoot–midfoot	*Sag*	−1.9	−3.0– –0.9	−0.2	−1.2– 0.7	3.3	2.3– 4.2	−7.7	−8.9– –6.5	4.8	3.9– 5.6	−7.7	−8.9– –6.5	7.8	6.6– 9.0
	*Frt*	3.9	2.8– 5.1	1.5	0.6– 2.5	−0.8	−1.8– 0.1	4.1	2.7– 5.4	6.2	5.2– 7.2	−5.7	−6.6– –4.8	−2.8	−5.2– –0.4
	*Trn*	−4.6	−5.4– –3.8	−2.6	−3.2– –1.9	−0.8	−1.8– 0.1	4.1	2.7– 5.4	3.4	2.8– 4.0	−8.1	−8.9– –7.3	4.0	2.8– 5.3
Medial forefoot–midfoot	*Sag*	−1.3	−2.6– –0.01	−0.5	−1.6– 0.6	3.7	2.5– 4.8	−8.5	−9.9– –7.1	4.7	3.5– 5.8	−9	−10.5– –7.5	8.2	7.0– 9.4
	*Frt*	−0.5	−1.4– 0.4	−1.4	−2.1– 0.6	−0.5	−1.2– 0.2	−1.9	−3.2– 0.8	1.6	0.8– 2.5	−3.9	−4.9– –3.0	3.1	2.1– 4.2
	*Trn*	1.8	1.1– 2.6	1.7	0.9– 2.4	1.6	0.8– 2.3	−3.7	−4.5– –2.8	2.9	2.2– 3.6	−5.4	−6.2– –4.6	6.5	5.9– 7.2
Hallux – medial forefoot	*Sag*	17.4	15.9– 19.0	12.7	11.4– 14.1	6.1	5.1– 7.2	33.2	31.2– 35.1	44.9	43.3– 46.7	0.5	−0.4– 1.4	−16.9	−18.4– –15.4
	*Frt*	0.9	−0.4– 2.3	0.9	−0.3– 2.1	0.01	−1.0– 1.0	−1.7	−3.5– 0.1	2.6	1.5– 3.7	−7.9	−9.5– –6.3	1.6	0.1– 3.0
	*Trn*	2.9	1.9– 3.9	1.9	1.1– 2.8	1.2	−2.0– −0.4	5.3	3.9– 6.6	6.1	4.8– 7.3	−2.8	−3.9– –1.8	3.1	1.1– 5.0

After heel strike there was plantarflexion at the calcaneus-tibia (0-5% gait) followed by dorsiflexion throughout mid stance (to 45%). The medial and lateral forefoot-midfoot segments and midfoot-calcaneus segments all dorsiflexed throughout stance up to approximately 45% of the gait cycle. There was marginally more motion between the two forefoot segments and the midfoot compared to the midfoot-calcaneus. The lateral forefoot segment showed more dorsiflexion earlier (0-10% gait) than the medial forefoot segment relative to the midfoot.

Between 50-65% of gait the calcaneus-tibia and medial and lateral forefoot to midfoot joints all plantarflexed, whilst the mean midfoot-calcaneus data suggests little movement. The hallux dorsiflexed whilst these other joints plantarflexed (40-60% gait) peaking at 40° just prior to toe off. During swing the hallux plantarflexed but remained in a dorsiflexed position, and all other joints showed some net dorsiflexion movement in preparation for next heel strike.

In the frontal plane the calcaneus-tibia and lateral forefoot-midfoot both displayed eversion after heel strike, up to 35-40% of gait. There was a small amount of eversion at the midfoot-calcaneus segment and inversion at both the medial forefoot-midfoot and hallux-medial forefoot segments between 10-40% of gait. These small movements were reversed between 40-60%. The calcaneus-tibia and lateral forefoot-midfoot inverted from 40-60% gait prior to toe off. In swing the calcaneus-tibia segment everted initially then inverted prior to heel strike (90-100% gait), there was otherwise very little movement.

In the transverse plane there was abduction at the calcaneus-tibia and lateral forefoot-midfoot segments between 0-10% of gait. The latter continued to abduct to 45% of gait, whereas the calcaneus-tibia reversed its motion to adduction between 10-60% of gait. Both medial and lateral forefoot segments adducted relative to the midfoot between 45-60% gait, whereas the hallux abducted during this same period. The mean data suggest there was negligible motion between midfoot-calcaneus. In swing there was abduction of the medial forefoot-midfoot segment throughout the period, but otherwise very little movement was observed.

The calcaneus-tibia and hallux-medial midfoot were the major contributors to sagittal plane function. Other joints contributed no more than 30% of all sagittal plane motion between 0-60% gait (i.e. stance). In 0-5% of gait these two joints contributed plantar flexion whereas all other joints were dorsiflexing. From 5-45% all joints except the hallux-medial forefoot contributed dorsiflexion. From 45 to 60% all joints contributed plantarflexion with the exception of the hallux-medial forefoot, which contributed dorsiflexion.

In the frontal plane the majority of joints contributed eversion between 0-15% of gait, and mainly inversion between 15-60% (i.e. until toe off). The calcaneus-tibia was a major contributor throughout gait, upto 40-50% of all frontal plane motion. The lateral forefoot-midfoot joint was the next major contributor, more so than both the medial forefoot-midfoot and midfoot-calcaneus joints. The latter contributed more during swing than in stance.

In the transverse plane the calcaneus-tibia made mainly large adduction contributions (10-60%) during stance, with some abduction earlier (0-10%). The lateral forefoot-midfoot joint contributed abduction from 0-45% of gait, and adduction up to toe off. The medial forefoot-midfoot joint made fluctuating adduction/abduction contributions. The smallest contribution was from the midfoot-calcaneus joint.

## Discussion

The kinematic data confirm that the normal pain free foot of those aged 18–45 is a highly compliant and multi articular mechanism whose function relies upon a range of contributions from all segments and movement in all three body planes. The greatest motion is in the sagittal plane which perhaps reflects the fact that ambulation seeks to maintain forward progression. Frontal and transverse plane movements were similar overall but consistently less than sagittal plane movements. Major sagittal plane contributions are from the ankle and sub talar joints (calcaneus-tibia in our data) and the hallux-medial forefoot joint. However, that sagittal plane movement at the lateral and medial forefoot-midfoot segments was larger than at the midfoot-calcaneus demonstrates the importance of forefoot joints.

There was greater movement proximally (calcaneus-tibia) and distally (medial/lateral forefoot-midfoot), compared to the central foot joints (talo-navicular, cancaneuo-cuboid, i.e. the midfoot-calcaneus joint in our data) in frontal and transverse planes (Table 1). This might perhaps reflect variable stiffness at different joints in the foot. It might equally reflect the fact that more distal and proximal bones are closer to the points of force application (calcaneus and metatarsal heads) and joint movement will tend to occur closer to the point of force application. However, that the contribution of the midfoot-calcaneus segment to overall foot behaviour was the least of any segment is a stark contrast to the focus on this joint in most clinical models of foot function. The data here suggest that this focus should shift to contributions from the lateral and medial forefoot segments relative to the midfoot.

Allowing for some differences in the segmental models used, the pattern and values of the foot kinematics we report are in line with prior reports [15,25,26]. Where apparent differences occur it seems clear that these are due to over simplification in prior models. For example, Legault *et al.* [25] combined all five metatarsals into a “forefoot” segment and reported its motion relative to the calcaneus. For the majority of stance there was almost no change in forefoot-calcaneus alignment in the frontal and transverse plane. This contrasts sharply with the ~12° of frontal and transverse plane motion in stance at the lateral forefoot-midfoot (max-min, Table 1), and equivalent Figures for the medial forefoot-midfoot. The difference in results could be due to contrasting directions of motion cancelling each other out in Legault *et al.* model, whereas in the model used in this work these separate motions are described. Furthermore, data in Bruening *et al.* [27] likewise suggests little or no frontal plane ‘midtarsal’ motion throughout much of stance (see their figure four). This could be incorrectly interpreted as meaning that there was no motion between any of the bones comprising their ‘forefoot’ (navicular, cuboid, cunieforms and all metatarsals) and the calcaneus. Thus, the foot between the metatarsal heads and calcaneus appears to be quite rigid (in the frontal plane). The data presented in this study, however, clearly contradicts this since we observed considerable motion between the structures that comprised the assumed rigid ‘forefoot segment’ defined by Breuning *et al.* [27]. This also dispels (see Okita *et al.* [28]) the long held clinical concept that the midtarsal joint ‘locks’ to provide a rigid lever [1] since all segments display compliance rather than rigidity during propulsion. These issues illustrate the pitfalls of violating the rigid body assumption in a multi-segment model. However, this also means that if the data reported here is to form a useful reference data set for future studies, it will only serve this purpose for studies whose foot model is the same or very close to the model we adopted.

The appropriate segmentation of the foot into relevant functional units is also critical to the validity of clinical models of foot function and we hope the data reported can inform these models in the future. We would argue that because the data presented illustrates the need for separation of the midfoot, lateral and medial forefoot segments, rather than combing these into a “forefoot” segment, a clinical model should do likewise. Furthermore, use of foot segment definitions that are common to experiments and clinical concepts will aid the transfer of knowledge from research into education and clinical practice.

Since so many foot joints share common ligament and muscle/tendon structures coupling between foot joints has already been explored as a means of simplifying and conceptually modelling the behaviour of the foot [29-31]. However, movements between the calcaneus-tibia and hallux-medial forefoot joints were synchronous after heel strike but asynchronous between 40-60% of gait, displaying large opposing motions. Furthermore, movement at the lateral and medial forefoot-midfoot segments was synchronous in the sagittal plane but asynchronous in the transverse and frontal planes. The lateral forefoot-midfoot and calcaneus-tibia movement patterns were very similar throughout stance in the frontal plane, but were synchronous only for 0-10% and 45-60% in the transverse plane. Whilst we present a descriptive rather than analytical investigation of coupling between foot joints, these data suggest that any coupling between foot joints is a transient characteristic, with planar and temporal variations during gait. Thus the binary concept of foot segments being coupled or not may fail to reflect the complexity of interrelationships between joints. More fundamentally, periods of asynchronous movement might simply be good evidence of no mechanical coupling at all, suggesting that periods of coupling have a motor control rather than mechanical basis. The factors governing the interrelationships between foot joints require further attention. Given the multi joint nature of all plantar soft tissues (except joint capsules) and that muscle contributions can be dynamic since they are controlled by the nervous system (whereas passive soft tissues offer entirely mechanical constraint), this is likely to be a complex problem.

Pronation and supination of the foot, and the associated “pronated” and “supinated” foot types, are popular terms used to simplify the combined movements of the multiple joints of the rear, mid and forefoot bones. However, it is often assumed that these concepts apply to individual joints or combinations of joints in the foot. The data here, however, reveal that joints are capable of complex combinations of frontal, transverse and sagittal plane motion and are rarely constrained to pronation and supination patterns.

Finally, a central feature of the prevailing clinical conceptual model of foot function is that all normal feet demonstrate the same movement profile [3] or that fairly binary criteria or concepts can be applied to all healthy feet [4]. The data presented here however, indicates that there are many different kinematic patterns in feet that are clinically normal i.e. symptom free. Whether the variation between individuals occurs more in one plane of motion, one joint or one phase of gait more than another, remains unresolved. Arguably, the nature of kinematic variation between individuals may be such that assuming average population data represents normal function is invalid. Indeed sub classifications of symptom free feet into “cavus” and “planus” foot type do reveal differences in structure and function [32-34]. The mechanical (i.e. anatomical) or neuromotor factors influencing normal variation between individuals in foot kinematics have not been fully explored, but these seem critical issues if our understanding of foot behaviour is to ever be considered complete.

Swing phase kinematics comprised mainly dorsiflexion (except for the hallux), eversion and abduction, especially at the lateral and medial forefoot-midfoot joints. These non-weight bearing movements are the result of complex interactions between active muscle forces (e.g. concentric action of anterior tibialis and long toe extensor muscles) and passive elastic forces, e.g. plantar flexion of the hallux after toe off due to elastic energy stored in the plantar facia and long toe flexor muscle/tendons during propulsion. As in this example, these passive and active forces may be antagonistic and rapid and accurate control of the active forces is a prerequisite for correct positioning of the foot during swing (to avoid hitting the other leg and floor) and to prepare for appropriate and safe ground contact in the next step. Deficits in this control, due to neuropathy or fatigue for example, may be a factor influencing slips and lateral ankle sprains that occur in the first milliseconds of ground contact.

There are several limitations to this work. The participants were pain free but this does not mean all feet were entirely free of changes in foot structure (e.g. sub clinical arthritic changes). However, in the absence of symptoms sub clinical changes might represent the real world normal foot population, which was the objective of this work. There were more females than males, which reflects the population sampled, but there is no strong evidence to suggest women have fundamentally different feet than men [35,36]. The cohort was relatively young but this reduces the influence of sub clinical structural changes such as arthritis, and changes associated with aging. Indeed, implicit in our inclusion criteria is the fact that the data are not intended to represent the healthy foot in older people. The multi-segment foot model chosen was developed from results of prior invasive and cadaver studies [8,37] and differs in some respects from others published. It has a different number and definition of segments in the forefoot for example, although the same or similar anatomical landmarks are used to locate markers and cluster plate positions. Reliability of such foot models has been very well discussed in prior literature [27,38-40], and since the anatomical landmarks and attachment approach we used is common to other models already reported, its reliability will not be notably different. It is worth noting that reports of foot model reliability include the effects of normal variability in gait kinematic data and standing position (used to establish 0°) due to collection of data in different sessions on the same day, and across different days. Thus, the actual contribution of the choice of foot model to the reported variation in kinematic data is less that the values reported in the literature. The minimal influence that the choice of foot model has on the reliability of foot kinematic data might help explain why there is as yet no report of an unreliable foot model.

Finally, the kinematic data were derived from barefoot walking and any basis for conceiving what constitutes a “normal foot” should include an appreciation of how footwear affects foot kinematics. Foot movement is a response to the loads applied to the foot and footwear fundamentally changes these loads. However, every shoe will affect the foot differently and so no single answer to this issue exists.

A final issue concerns the different ways to define planes in which the joint motions are reported. We chose to align the segment local co-ordinate axes to those of the leg during quiet standing. This avoids the as yet unresolved difficulty that some foot segments (e.g. talus, navicular, cuboid) do not have natural osseous axes that are easily and repeatedly identifiable using external landmarks. However, our approach removes any difference between participants in the absolute angular alignment of bones relative to each other. This could be important to describe different clinical foot types or pre and post-surgery evaluation. However, some of these differences might be better measured statically using surface scanning techniques, especially if the interest is in altered foot posture rather than motion. If changes in movement and position of joints during gait is the intended outcome, then defining more anatomically relevant axes is warranted. But to derive meaningful differences between groups of individuals (e.g. foot types) or experimental conditions (pre/post surgery), requires that markers are attached to the same anatomical locations on different people or different days with positioning errors smaller than the differences being investigated. It also assumes that the anatomical alignments achieved define planes of motion that are clinically relevant. For example, aligning the anterior/posterior axis of a co-ordinate system to the long axis of the first and fifth metatarsal seems possible using external landmarks, and this could provide useful measure of sagittal plane metatarsal position. However, alignment to the frontal and transverse plane angle of a metatarsal seems difficult since the landmarks for these are internal.

## Conclusion

This work sought to provide a comprehensive description of normal foot kinematics in a large population, using an appropriate number of foot segments to characterise foot behaviour, and including stance and swing phases. The data reveal the foot is a multiarticular structure, movements are complex and multiplanar, show incomplete evidence of coupling, and person to person variation that is as yet unexplained.
